# Collagen crosslink excretion and staging of oral cancer

**DOI:** 10.1038/sj.bjc.6600873

**Published:** 2003-04-01

**Authors:** I N G Springer, H Terheyden, A Dunsche, N Czech, M A A Suhr, M Tiemann, J Hedderich, Y Açil

**Affiliations:** 1 Department of Oral and Maxillofacial Surgery, University of Kiel, Arnold-Hellerstr 16, D-24105 Kiel, Germany; 2 Department of Nuclear Medicine, University of Kiel, Arnold-Heller Straße 9, D-24105 Kiel, Germany; 3 Department of Oral and Maxillofacial Surgery, Klinikum Nord, Tangstedter Landstrasse 400, 22417 Hamburg, Germany; 4 Department of Surgical Pathology, University of Kiel, Niemannsweg 11, 24105 Kiel, Germany; 5 Institute of Medical Informatics and Statistics, University of Kiel, Brunswiker Str. 10, 24105 Kiel, Germany

**Keywords:** pyridinoline, collagen crosslinks, staging, oral squamous cell carcinoma, bone infiltration

## Abstract

Lysylpyridinoline (LP) and hydroxylysylpyridinoline (HP) are collagen crosslink residues of which the urinary concentration reflects the level of connective-tissue turnover. HP is ubiquitous in tissue, whereas LP is specific for bone. The purpose of this investigation was to assess the sensitivity and specificity of an increased urinary concentration of both HP and LP in indicating infiltration of mandibular bone by an oral squamous cell carcinoma (OSCC) or recurrence of the disease after successful therapy. We investigated the history and urine levels in 116 adult patients, who were divided into the following groups. Group 1: patients with OSCC with bone infiltration (*n*=17); group 2: patients with confirmed OSCC (*n*=12) without evidence of bone infiltration; group 3: patients with recurrence of an OSCC (*n*=13); group 4: patients without clinical evidence of disease (*n*=74). The range and upper limit of normal values (HP_max_ and LP_max_) were measured from the normal controls in group 4. Levels of LP and HP were measured by HPLC and fluorescence detection. There was a significant difference in the average urinary levels of LP and HP between groups 1–4 (*P*<0.001). The presence of mandibular bone infiltration could be detected with a sensitivity and specificity of 100% when comparing groups 1 and 2. Presence of tumour tissue could be detected with a sensitivity of 90%. In conclusion, a normal LP concentration in patients with an OSCC strongly suggests that bone invasion by the disease has not taken place. If both urinary HP and LP are elevated, disease recurrence is highly likely.

Turnover and resorption of collagen in tissues adjacent to malignant disease processes may be increased (Body and Delmas, 1992). Hydroxylysylpyridinoline (HP) and lysylpyridinoline (LP) are two nonreducible crosslinks of mature collagen, which are formed by a sequence of post-translational modifications. Hydroxy-lyslpyridindine is a derivative of three residues of hydroxylysine and is present in virtually all mature tissues (tendon, vessel wall, cartilage, dentine and bone). Lysylpyridindine is a derivative of two residues of hydroxylysine and one residue of lysine and is found principally in dentine and bone. (Body and Delmas, 1992; Eyre, 1992; Miyamoto *et al*, 1994; Acil *et al*, 1996,^2000^; Papatheofanis, 1997; Tamada *et al*, 2001). It has been suggested that the detection of LP in the serum or urine may be a helpful marker in establishing and possibly quantifying bone matrix resorption (Body and Delmas, 1992; Miyamoto *et al*, 1994; Vinholes *et al*, 1996; Papatheofanis, 1997; Tamada *et al*, 2001). The measurement of HP and, in particular, LP has been shown to be valuable in detecting the presence and/or extent of bony metastases in patients with multiple myeloma, carcinoma of the breast, lung, prostate gland, kidney, throat and digestive tract (Takeuchi *et al*, 1996; Papatheofanis, 1997; Walne *et al*, 1997; Yoshida *et al*, 1997; Coleman *et al*, 1999; Marttunen *et al*, 1999; Tamura *et al*, 1999; Woitge *et al*, 1999,^2001^; Demers *et al*, 2000; Fontana and Delmas, 2000; Liubimova *et al*, 2000; Izumi *et al*, 2001; Tamada *et al*, 2001).

Treatment decisions in oral cancer are influenced by the infiltration of mandibular bone, with consequently increased operating time and extent of resection, where surgery is indicated (Reichart and Philipsen, 1999; Baker *et al*, 2001; Hong *et al*, 2001; Schimmele, 2001). Computertomography, fluorodeoxyglucose (FDG) positron emission tomography (PET) or technetium 99 m methylene diphosphonate (MDP) single-photon emission computed tomography (SPECT) are the methods of choice in assessing bony involvement of malignant disease (Minn *et al*, 1993; Huntley *et al*, 1996; Schimming *et al*, 2001). Measurement of the concentration of LP in the urine may be an alternative. Oral squamous cell carcinoma (OSCC) may result in increased concentrations of HP in the urine, whether mandibular bone has been infiltrated or not. We suggest that the measurement of HP and LP in the urine of recall patients after apparently successful treatment for oral cancer may assist in the screening for recurrence. We discuss the role of collagen crosslinks as markers of tissue turnover in their potential role in the staging of oral cancer.

## MATERIALS AND METHODS

Patients were recruited from the Tumour Clinic in the Department of Oral and Maxillofacial Surgery, University of Kiel, Germany. In all, 475 urine samples from 116 patients (age range 36–91 years) were analysed. A prospective double-blind study was designed, and the patients divided into four groups as follows:

*Group 1 (n*=*17)*: patients with OSCC (pT4 N1-2 M0) with bone infiltration (10 males, seven females; eight patients 39–60 years of age; nine patients 61–88 years of age).

*Group 2 (n*=*12):* patients with OSCC (pT1-3 N0-1) with no bone infiltration (seven males, five females, six patients 44–60 years of age; six patients 61–81 years of age).

*Group 3 (n*=*13):* patients with recurrence of the disease, in seven of whom (*group 3b*) we obtained samples up to 6 months prior to the confirmation of the recurrence (nine males, four females; four patients 52–60 years of age; nine patients 61–83 years of age).

*Group 4 (n*=*74):* patients without disease (53 males, 21 females; 28 patients 36–60 years of age; 56 patients 61–91 years of age), control group.

Patients who were seen in our follow-up programme after apparently adequate initial treatment of OSCC were used as controls if treatment had been completed more than a year prior to entry into the study. In this group, an R_0_-resection and <T2-disease was required for inclusion as a control. Patients who had a history of malignancy other than OSCC or had alterations in renal function (urea>50 mg dl^−1^, creatinine>1.2 mg dl^−1^) were excluded. Recall patients were excluded if a previous recurrence had been documented and successfully treated. Patients were also excluded if they had had a surgical procedure or a trauma less than 6 months prior to entry into the study (see results). Patients of groups 1–3 were staged by ultrasound and computed tomography. Technetium 99 m MDP bone scans with planar imaging and SPECTs were additionally performed on all patients included in the study. A chest X-ray, abdominal ultrasound, endoscopic examination of the upper aerodigestive tract and gynaecological examinations in female patients completed the assessment of concomitant disease processes. The histopathological examination was performed by a single pathologist.

### Preparation and hydrolysis of urine

Samples were taken in the morning and stored at −70°C until further processing. All laboratory investigations were performed in a single laboratory. Hydroxylyslpyridinoline and LP levels seem to be stable in urine samples for over 10 years if samples are stored at this temperature. The urine samples were centrifuged at 1000 r.p.m. for 5 min. A measure of 2 ml of supernatant was lyophilized and subsequently redissolved in 2 ml 6 N hydrochloric acid. The samples were hydrolysed at 110°C for 24 h and centrifuged at 1000 r.p.m. for 5 min. A volume of 1 ml of each hydrolysate was added to a mixture of 1 ml glacial acetic acid, 2 ml *n*-butan-1-ol and 5 ml 10% CF-1-slurry (fibrous cellulose powder, Whatman, Maidstone, UK). The CF-1-slurry was composed of 10% (w v^−1^) CF-1 in a mobile phase containing *n*-butan-1-ol, glacial acetic acid and water (4 : 1 : 1). A column was prepared by adding the mixture of hydrolysate and CF-1-slurry as described above to an econo-column polyprop (40 × 8 mm, Bio-Rad München, Germany). The resin was washed three times with 5 ml of the mobile phase. Subsequently, the pyridinium-containing eluate was eluted from the column with 3 × 2 ml distilled water into a 15 ml plastic tube and traces of *n*-butan-1-ol were removed from the surface of the eluate. Thereafter, the lyophylised eluate was redissolved in 1 ml 0.22% (v v^−1^) *n*-heptafluorobutyric acid (HFBA) and centrifuged at 1000 r.p.m. for 5 min. A volume of 200 *μ*l of the sample was analysed by HPLC (as below). The variations within and between series were 2 and 4.8%, respectively.

### Pyridinoline standards

The HPs and LPs were quantified by external standards gained from a commercially available adult bovine bone gelatin (Deutsche Gelatine-Fabriken Stoess, Eberbach/Baden, Germany) prior to the application of the samples to the chromatography system. Hydroxylyslpyridinoline and LP were purified by a preparative reverse-phase-column HPLC and the degree of purity was verified by amino-acid analysis (>98% of dry weight) according to a method previously described (Acil and Müller, 1994; Acil *et al*, 1996,^2000^) Serial dilutions of HP between 0 and 2250 (pmol nmol^−1^) and LP 0 and 1200 (pmol ml^−1^) were analysed to demonstrate the linear response of the external standards.

### Analysis of HP and LP by reverse-phase-column HPLC

Chromatography was performed on a Dionex HPLC system (Idstein, Germany) at 22°C. The flow rate was 0.7 ml min^−1^ using two continually degassed solvents: (A) 0.22% (v v^−1^) *n*- HFBA in water and (B) 0.22% (v v^−1^) HFBA in 80% (v v^−1^) acetonitrile. The resin (Inertsil ODS-3 5 *μ*m, 125 × 4.6 mm C_18_) was equilibrated with 18/82% solvent B to solvent A prior to the application of the sample (200 *μ*l in 0.22% (v v^−1^) HFBA in water). The column was washed with 18/82% (v v^−1^; B/A) for 5 min and developed with the following step gradients:

1. 18–20% solvent B over 20 min; the peaks of HP and LP were eluted at approximately 18 and 20 min;
2. 20–25% solvent B in 4 min;
3. 25–100% solvent B in 1 min plus washing of the column for another 5 min with 100% solvent B;
4. 100–18% over solvent B in 4 min; 1 min was used for column equilibration, thereafter. The next sample was injected after 35 min.

Fluorescence measurements were obtained with an excitation wavelength of 297 nm and emission wavelength of 397 nm and the concentrations of HP and LP expressed in pmol ml^−1^. After dilution (1 : 20) of 1 ml of patient urine, the urinary creatinine content was measured by the colorimetric Jaffé-reaction and expressed in mg dl^−1^ (Beckman Creatinine Analyzer 2, USA). The urinary content of HP and LP was expressed in relation to the urinary creatinine concentration, that is, in nmol mmol^−1^ creatinine.

### Statistical methods

**Figure 1:**
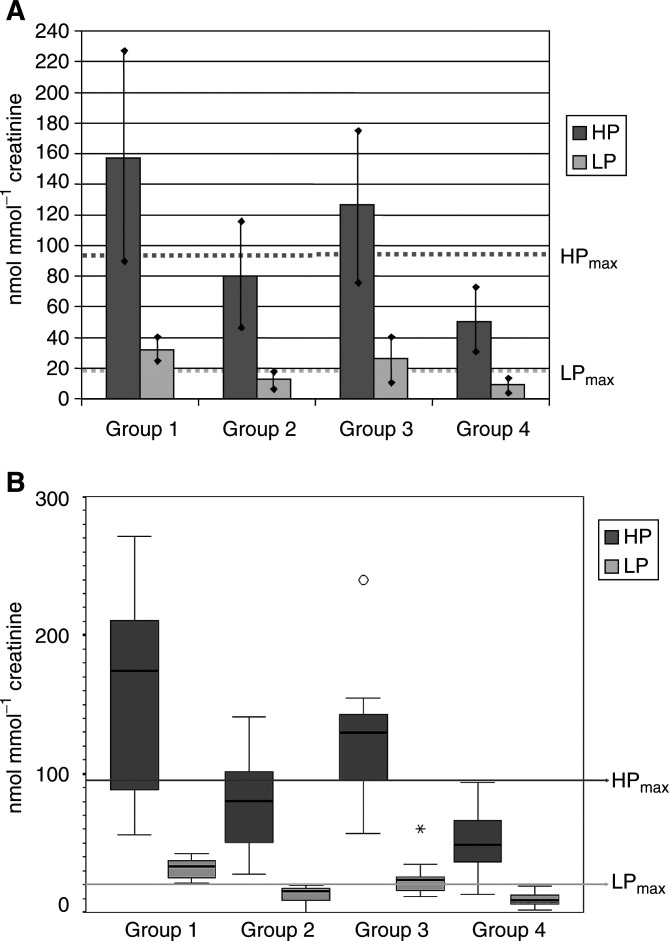
(**A**) Arithmetic means of concentrations of urinary HP and LP: in groups 1 (OSCC with mandibular bone infiltration, *n*=17), 2 (OSCC without mandibular bone infiltration, *n*=12), 3 (recurrence of OSCC, *n*=13) and 4 (control group, *n*=74). Dotted lines mark HP_max_ (95 nmol mmol^−1^ creatinine) and LP_max_ (20 nmol mmol^−1^ creatinine). The urinary levels of HP in groups 1–3 are significantly different from group 4. The urinary concentrations of LP in groups 1 and 3 are significantly different from group 4. Moreover, in groups 1 and 3, the average urinary content of HP and LP exceeded HP_max_ and LP_max_ significantly. Please note that in group 2, HP is significantly elevated as a marker of increased tissue turnover as compared to group 4, but does not exceed HP_max_. Length of vertical bars: standard deviation times 1.96. (**B**) Medians of concentrations of urinary HP and LP: Each box shows the median, quartiles and extreme values of groups 1 (OSCC with mandibular bone infiltration, *n*=17), 2 (OSCC without mandibular bone infiltration, *n*=12), 3 (recurrence of OSCC, *n*=13) and 4 (control group, *n*=74). Lines mark HP_max_ (95 nmol mmol^−1^ creatinine) and LP_max_ (20 nmol mmol^−1^ creatinine). The urinary levels of HP in groups 1–3 are significantly different from group 4. The urinary concentrations of LP in groups 1 and 3 are significantly different from group 4. Moreover, in groups 1 and 3, the urinary content of HP and LP exceeded HP_max_ and LP_max_, significantly. As regards groups 1 and 2, the LP_max_ line is separating LP values, completely. O and * label values of one patient, which significantly exceeded the normal range in the recurrence group.

There was no statistically significant difference between the sexes, and a normal distribution of the urinary concentrations of HP and LP was seen in groups 1–4. An analysis of variance (ANOVA) was performed to evaluate statistically significant differences between groups 1–4. Multiple comparisons were performed according to Games-Howell. Since the variance of groups 1–4 appeared to be not strictly homogeneous (Figure 1A and B

), medians were calculated and the significance of differences tested according to Kruskal–Wallis to confirm results obtained by the ANOVA. The arithmetic mean of the urinary levels of HP and LP of the control group (group 4) was calculated and 1.96 standard deviations used to define the normal range. These upper limits were called HP_max_ and LP_max_. The urinary concentration beyond HP_max_ and LP_max_ was elevated with a *P*<0.025. The sensitivity and specificity in respect of the differentiation of groups 1 (OSCC with bone infiltration) and 2 (OSCC without bone infiltration) based on HP_max_ and LP_max_ as cutoff points were calculated. The suspicion of mandibular bone infiltration on the basis of increased values of HP or LP that could not be confirmed by diagnostic measures such as those described above (first part of the Materials and Methods section) was classified as being false positive. Similarly, increased values of HP and LP in patients without tumour recurrence were classified as being false positive. Any indication of mandibular bone infiltration by these same diagnostic measures, without increased values of HP or LP, was classified as being false negative. Normal levels of HP and LP in the presence of a confirmed recurrence was also classified as being false negative.

In seven out of 13 patients in group 3, samples had been obtained 6 months prior to the recurrence (group 3b). For these seven patients, a paired *t*-test was performed taking both the former and the current value into account. The sensitivity and specificity in detecting the presence of OSCC tissue was evaluated. The urinary concentrations of HP and LP in the patients of groups 1–3 were compared to the urinary concentrations of HP and LP of the patients of group 4 and a receiver operator characteristic curve (ROC curve) constructed.

### Ethics

The study was conducted in accordance with the standards of the Ethics Committee of the University of Kiel (chairman: Jürgen Schaub, MD, PhD, Professor for Pediatrics, Head of the Department of Pediatrics, University of Kiel, Germany; registration number of the present study: AZ D 309/01) and with the Helsinki Declaration of 1983. The patients were informed about the aim and design of the study and written consent was obtained.

## RESULTS

In all, 475 urine samples from 116 patients were analysed in the course of the present study. The average urinary levels of HP and LP in nmol mmol^−1^ creatinine are graphically represented in Figure 1A and B. The standard deviation is shown in brackets, and the ratio of HP:LP given as follows:

*Group 1:* HP: 157.3 (68.9); LP: 31.6 (7.5); HP/LP: 4.9 (1.7);
*Group 2:* HP: 80.0 (35.4); LP: 12.9 (9.9); HP/LP: 5.5 (2.1);
*Group 3:* HP: 119.7 (47.0); LP: 23.5 (12.2); HP/LP: 5.6 (2.4);
*Group 3b:* HP: 59.2 (20.1); LP: 11.4 (3.4); HP/LP: 5.2 (0.85).
*Group 4:* HP: 50.5 (20.7); LP: 9.5 (4.8); HP/LP: 6.0 (2.7).

**Figure 2:**
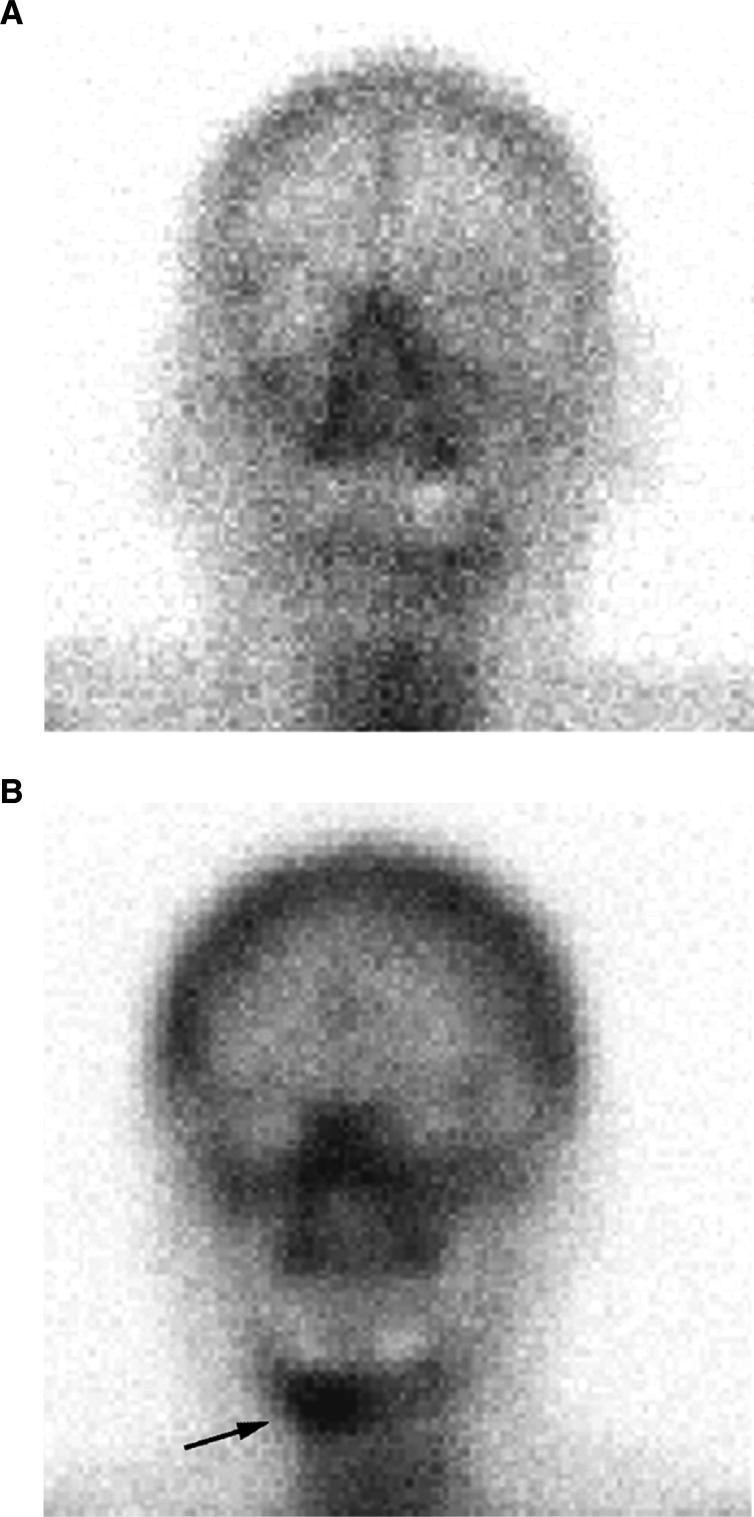
(**A**,**B**) Scintigraphy of a patient with an OSCC with (**A**) and without (**B**) infiltration of the mandibular bone. Technetium 99m MDP bone scans with planar imaging and SPECT were performed in the course of the staging. No increased activity is visible in (**A**), but a lesion of the right mandible is seen in (**B**) (arrow). For the chromatograms of these patients see Figure 3A and B.

HP_max_ was 95 nmol mmol^−1^ creatinine and LP_max_ 20 nmol mmol^−1^ creatinine. Differences between the average concentrations of HP and LP of groups 1–4 were statistically significant (ANOVA: *P*<0.001, Kruskal–Wallis: *P*<0.001). The average urinary HP and LP concentrations were significantly elevated in group 1 compared to group 2 (*P*<0.001 for HP and LP), in group 1 compared to group 4 (*P*<0.001 for HP and LP) and in group 3 compared to group 4 (*P*=0.001 for HP and *P*<0.01 for LP) (Figures 1, 2

and 3

**Figure 3:**
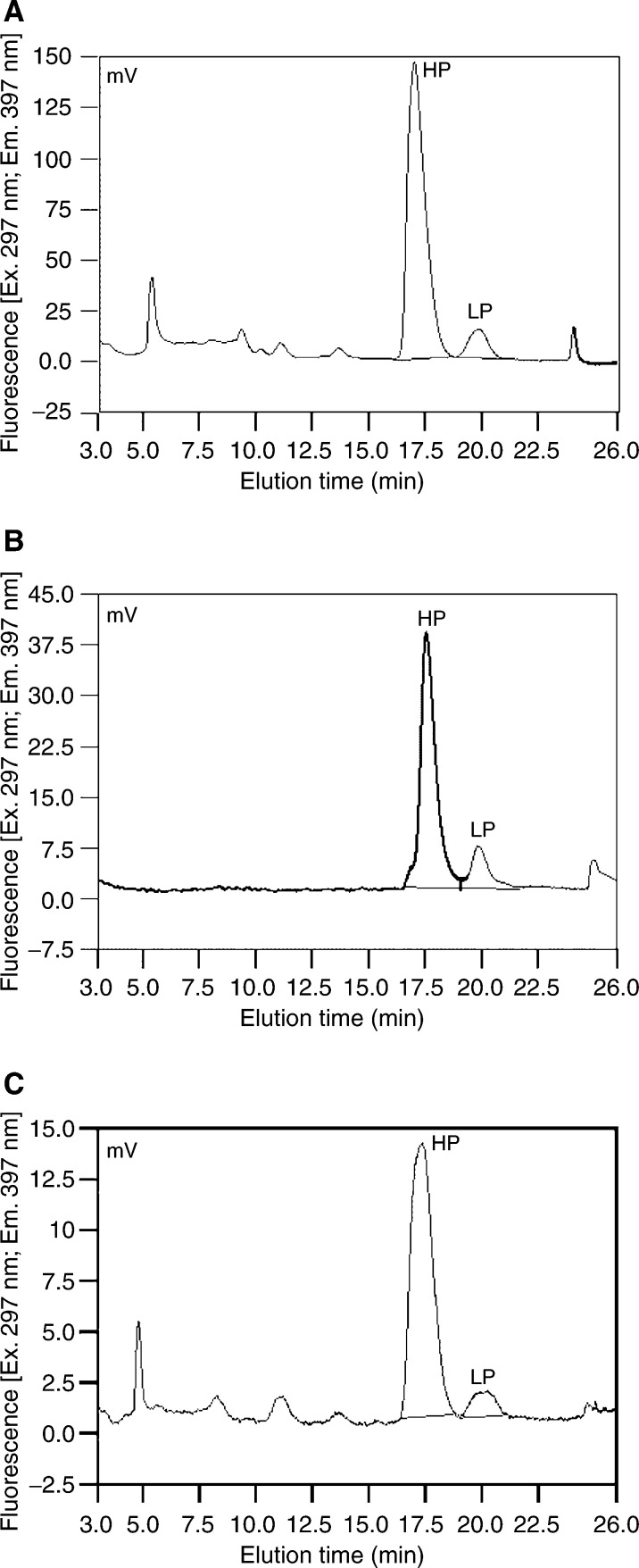
(**A–C**) Chromatograms of a patient with OSCC with (**A**) and without (**B**) infiltration of the mandibular bone and a patient with no cancer disease (**C**). The fluorescence was monitored with excitation at 297 nm and emission at 397 nm. The HP peak arose at 17.5 min after injection, followed by the LP peak. Please note the increased HP and LP peaks in (**B**) compared to (**A**). Please note the low HP and LP levels of the patient without current evidence of tumour (**C**). For the scintigraphies of patients (**A**) and (**B**) see Figure 2A and B. Please note that the peaks appear to be of the same size, but that the scale is different.

). The average urinary HP concentration in group 2 was significantly elevated compared to group 4 (*P*<0.05). When comparing the average HP/LP ratio, it was found to be significantly lower in group 1 compared to group 4 (*P*<0.05). No significant differences were found between the average HP/LP ratios of the other groups. A significant increase was found for HP (*P*=0.003) and LP (*P*=0.02) at the time of the recurrence when comparing group 3 with group 3b.

The average urinary concentrations of both HP and LP were elevated in groups 1 and 3 (*P*<0.025). The average urinary content of HP and LP of group 2 was less than HP_max_ and LP_max_ (Figure 1A, B). At this level of HP_max_, measurement of HP had a sensitivity of 61.5% and a specificity of 76.6% in separating groups 1 and 2. LP_max_ at the cutoff points chosen had a sensitivity and a specificity of 100% for separating groups 1 and 2. Sensitivity and specificity of the present assay in regard to the detection of the presence of OSCC tissue (groups 1–3 *vs* group 4) was evaluated. The ROC curve showed a cutoff of 55.7 nmol mmol^−1^ creatinine for HP with a sensitivity of 90% and a specificity of 63%, and a cutoff of 11.15 nmol mmol^−1^ creatinine for LP with a sensitivity of 90% and specificity of 69%.

## DISCUSSION

We intended to assess the ability of HP and LP as urinary markers of connective tissue and bone turnover in patients with OSCC in detecting bony invasion by malignant disease, as well as serving as a marker for tumour recurrence. We were able to show that the measurement of LP in the urine could separate groups 1 (OSCC with bone infiltration) and 2 (OSCC without bone infiltration) with a sensitivity of 100% and a specificity of 100% while no false negatives or false positives were observed. We suggest that when the level of LP exceeds LP_max_ in a patient with a confirmed OSCC, bony invasion by the malignant process is highly likely and further investigation is required. We furthermore suggest that a urinary level of LP less than LP_max_ in patients with OSCC may obviate the need for further investigations. The measurement of both LP and HP in the urine is recommended for the detection of bony metastases in a multitude of malignancies, indicating a higher specificity of LP for bone turnover, as compared to HP alone (Pecherstorfer *et al*, 1995; Takeuchi *et al*, 1996; Papatheofanis, 1997; Walne *et al*, 1997; Coleman *et al*, 1999; Marttunen *et al*, 1999; Tamura *et al*, 1999; Woitge *et al*, 1999,^2001^; Demers *et al*, 2000; Liubimova *et al*, 2000; Tamada *et al*, 2001). Urinary HP is not specific for bony invasion, but may nevertheless be increased in the urine of patients with bony metastases as compared to patients without bony metastases. Increased HP values in patients with bony invasion may be related to the presence of tumour tissue alone. This assumption is based on the fact that the average urinary HP concentration was significantly higher in patients with OSCC without mandibular bone invasion (group 2) as compared to the control group. Urinary LP was not significantly increased in patients of group 2 as compared to patients of group 4 further indicating specificity for bone turnover. It has been shown that neonatal rat aortic smooth muscle cells produce detectable amounts of HP (Stone *et al*, 1992), indicating that HP is synthesised in blood vessel walls.

The average urinary concentration of HP in group 2 did not exceed HP_max_, although the arithmetic mean of the urinary concentration of HP in group 2 is significantly different from the average urinary concentration of HP in the control group. Therefore, the urinary levels of HP were not able to separate group 2 from group 4. The urinary HP may be below HP_max_ in a patient with an active malignant process (see below). We nonetheless suggest that the measurement of HP in the urine may be helpful in screening for tumour recurrence.

Groups 3 and 4 could be separated by the measurement of urinary HP and LP, since average urinary concentrations exceeded HP_max_ and LP_max_. The differences in the arithmetic means of HP and LP of group 3 as compared to group 4 were highly significant. We suggest that cancer in the patient history does not preclude that patient from being used as a control (group 4) if the tumour was smaller than T2 in size, was successfully treated and showed no signs of recurrence. If we had compared group 3 with a control group with no cancer in the patient history, the difference might have been even greater than observed in this study. However, we suggest that this may not be the case in the present study and that group 4 (control group) may have served as a reliable control group in this paper leading to HP_max_ and LP_max_ values that could separate groups 1 (bone infiltration) and 2 (no bone infiltration) with a sensitivity and a specificity of 100%.

In this study, the increased turnover of collagen was indicative of tumour progression. Calculation of the ROC curve revealed a sensitivity of 90% for the presence of tumour progression if the cutoff is chosen at 55.7 nmol mmol^−1^ creatinine for HP and at 11.15 nmol mmol^−1^ creatinine for LP. It is suggested that the measurement of LP and HP in the urine of patients after treatment of cancer may be used in the course of recall examinations with the ultimate goal of increasing the chances of early detection of a recurrence. Patients in groups 3 and 3b indicate that periodic monitoring of the collagen crosslink excretion may be useful in establishing individual trends in relation to the baseline value of HP and LP excretion. Increased values during the first 6 months following surgery may not indicate relapse if the trend is stable or decreasing. Normal values may indicate a recurrence if the levels increase. If the collagen crosslink excretion increases in recall patients, recurrence or metastasis is strongly suggested, unless other reasons for an increase in tissue turnover are found.

In the course of this part of the study, we were looking at group 3 with no special interest as to whether bone was infiltrated by the disease or not. In fact, in a large fraction of these patients bone infiltration was present. However, we were interested in HP and LP as an aid in detecting patients in cancer recall hours with increased tissue resorption.

In all, 105 further patients, in whom urinary levels were obtained, had to be excluded from the study on the basis of the exclusion criteria stated above. Of these 105 excluded patients, 33 (31.4%) had elevated concentrations of HP and LP beyond our suggested levels of HP_max_ and LP_max_. In total, 17.6% (*n*=39/(105+116)) of the recall patients were false positive, meaning that their HP and LP were greater than Hp_max_ and LP_max_ but there was no evidence to confirm the presence of a recurrence. We suggest that this should nonetheless at least alert the clinician to the possibility of a recurrence. Of these false-positive samples, 66.7% (*n*=26/39) may have been because of a surgical procedure performed less than 6 months prior to obtaining the sample of urine, whereas 33.3% of the false-positive samples (*n*=13/39) were because of the presence of active sites of inflammation. Seven of these 13 patients had confirmed osteomyelitis of the mandibular bone without evidence of tumour recurrence. No false-negative results were obtained, meaning that there were no patients with normal values where a recurrence was subsequently documented. The follow-up period has been greater than 6 months for all recall patients.

The 17.6% false-positive rate in the recall patients excluded from the study was because of surgical procedures having been performed in the 6 months prior to the investigation, or inflammatory disease such as osteomyelitis. Other possible causes for false positives are trauma, further malignancy or bone disease such as osteoporosis, rheumatoid arthritis, primary hyperparathyroidism, active Paget's disease of the bone, Ehlers–Danlos syndrome type VI and others (Eyre, 1992; Miyamoto *et al*, 1994; Acil *et al*, 1995; Chen *et al*, 1996; Coleman *et al*, 1999; Woitge *et al*, 1999). Urinary HP and LP are valuable trend markers of diseases with altered bone resorption as well as their treatment (Eyre, 1992; Acil *et al*, 1996; Coleman *et al*, 1997; Liubimova *et al*, 2000). Osteomyelitis may be added to this list following this study, since it led to a significant amount of false positives in the group of our recall patients (seven out of 39 false positives). The study has been continued monitoring the course and treatment response of mandibular osteomyelitis.

The cost of detection of urinary HP and LP is low when performed in a clinical laboratory on a routine basis. Efforts are continually being sought to detect bony invasion by OSCC (Minn *et al*, 1993; Huntley *et al*, 1996; Baker *et al*, 2001; Hong *et al*, 2001; Schimmele, 2001; Schimming *et al*, 2001). Clinical examination, ultrasonography and the analysis of a urine sample may provide sufficient information for treatment planning, reducing the exposure of the patient to radiation.
